# Preparation of HCPT-Loaded Nanoneedles with Pointed Ends for Highly Efficient Cancer Chemotherapy

**DOI:** 10.1186/s11671-016-1491-9

**Published:** 2016-06-14

**Authors:** Shichao Wu, Xiangrui Yang, Yang Li, Hongjie Wu, Yu Huang, Liya Xie, Ying Zhang, Zhenqing Hou, Xiangyang Liu

**Affiliations:** Institute of Soft Matter and Biomimetics, College of Materials, Xiamen University, Xiamen, 361005 China; Department of Chemistry, College of Chemistry and Chemical Engineering, Xiamen University, Xiamen, 361005 China; School of Pharmaceutical Sciences, Xiamen University, Xiamen, 361005 China; The First Affiliated Hospital of Xiamen University, Xiamen, 361002 China; Department of Radiology, Taishan Medical University, Tai‘an, China

**Keywords:** High-aspect ratio, Pointed-end, HCPT, Cellular uptake

## Abstract

The high-aspect-ratio nanoparticles were proved to be internalized much more rapidly and efficiently by cancer cells than the nanoparticles with an equal aspect ratio. Herein, a kind of high-aspect ratio, pointed-end nanoneedles (NDs) with a high drug loading (15.04 %) and the prolonged drug release profile were fabricated with an anti-tumor drug—10-hydroxycamptothecin (HCPT)—via an ultrasound-assisted emulsion crystallization technique. It is surprising to see that the cellular internalization of NDs with an average length of 5 μm and an aspect ratio of about 12:1 was even much faster and higher than that of nanorods with the same size and the nanospheres with a much smaller size of 150 nm. The results further validated that cellular internalization of the nanoparticles exhibited a strong shape-dependent effect, and cellular uptake may favor the particles with sharp ends as well as a high-aspect ratio instead of particle size. The NDs with enhanced cytotoxicity would lead to a promising sustained local drug delivery system for highly efficient anticancer therapy. More importantly, the fabrication of NDs opens a door to design new formulations of nanoneedle drug delivery systems for highly efficient cancer.

**Electronic supplementary material:**

The online version of this article (doi:10.1186/s11671-016-1491-9) contains supplementary material, which is available to authorized users.

## Background

Cancer, causing about eight million deaths/year [1], has become the most common factor in worldwide mortality. Chemotherapy is one of the most commonly used strategies in cancer therapy, but their efficacy is largely limited by suboptimal pharmacokinetic properties, such as low stability, poor absorption, toxicity, distribution, and elimination [2]. Therefore, over the decade, the concentration of research was focused on improving the efficacy of the chemotherapy. In this regard, some novel drug delivery systems, such as microspheres, nanoparticles, and liposomes, have been proposed. It follows that mutifunctional particles with long-circulating [3, 4], targeting [5–7], imaging [8, 9], and pH-sensitivity [10, 11] have been developed, which further improve their pharmacokinetic properties.

The cellular uptake of the particles is an important factor to the therapeutic effect of the drug delivery systems. To facilitate the cellular uptake, the concentration has been focused on controlling the size of the particles. It is formerly accepted that the size of nanoparticles may play a paramount role in cellular uptake [12–17], although its effect on the uptake into non-phagocytic cells is still not fully understood. It is believed that the upper limit of the size of the particles that could be internalized into non-phagocytic cells via non-specific endocytosis was 150 nm [18–21]. Rejman et al. found that spherical nanoparticles, with a size of up to 500 nm, could be taken up by non-phagocytic cells, whereas spherical particles with a diameter of 1 μm could not [22]. As the size increases, the amount and the speed of the internalization of the beads were found to be significantly reduced compared with the 50-nm beads [22]. He et al. came to the similar conclusion that smaller particles were more likely to be uptake by non-phagocytic cells [23]. As to the particles smaller than 100 nm, there would be an optimum size of 50 nm for cellular uptake [23]. According to the aforementioned studies and others’ work [12, 24–27], the non-phagocytic cellular uptake shows a clear trend: the uptake.

We notice that all these studies are performed using spherical nanoparticles and neglect the influence of particle shape. This is most likely due to the lack of easy-to-use methods available to control the particle shape. Since the last decade, the non-spherical particle shape is attracting more and more attention for their potential effect on drug delivery, due to the progress made in attaining diversely shaped particles [28–33]. Although the effect of shape on drug delivery has not been thoroughly examined, there has already been evidence showing that many properties of the particles, the degradation, the biodistribution, and the cellular internalization of the particles, will be particle shape dependent [34–40]. Non-spherical particles have already been identified to possess particular pharmacokinetic properties which may be more favorable towards the therapeutic intent [41]. Non-spherical particles with asymmetric shapes could offer unique degradation profiles, since the particle shapes are changing over time [38]. Zero-order release, the goal of many sustained drug delivery systems, was achieved with a hemi-spherical particle which only allowed degradation on the surface. Non-spherical particles have also been found to exhibit distinct in vivo distribution profiles, which may provide a new means for targeting of specific organs or tissues, such as the spleen [42], the lungs [43], and tumor tissues [44, 45]. And above all, shape plays an important role in the cellular internalization, hence largely affecting the treatment result. Because when the particles reach the target tumor site, by systemic administration or direct local administration, they must be able to enter cancer cells to reach the therapeutic targets within the cells and kill them. In recent studies, it was found that the cancer cells preferred particles with a high-aspect ratio. Nanorods were found to penetrate tumor cells more rapidly than nanospheres, perhaps due to improved transport through pores [46]. The cellular uptake by A375 human melanoma cells increases in number and speed with the aspect ratio of mesoporous silica nanoparticles of constant diameter [47]. In another systematic study, the effect of the particle shape of cationic, cross-linked, and PEG-based hydrogel particles on cellular uptake was examined with HeLa cells [48]. It is found that the cubic particles with a size of 3 um could be internalized by HeLa cells, although it was only limited. Interestingly, the internalization of the rod-shaped, high-aspect-ratio nanoparticles occurred to be with much higher efficacy than that of nanoparticles with an equal aspect ratio.

Although, the result, that the high-aspect ratio could increase the cellular uptake, has been proven by more and more studies, few reports indicated that it has been used in the drug delivery system. According to the aforementioned research results, we could infer that anticancer drug-loaded nanoparticles with a high-aspect ratio perhaps show an enhanced effect in cancer cell intake, which could optimize the efficiency of drug delivery, and eventually benefit the cancer therapy. However, not many reports were found about the property of the pointed-end non-spherical particles. Hence, in this study, we will fabricate the high-aspect ratio, pointed-end, and 10-hydroxycamptothecin (HCPT)-loaded nanoneedles (NDs) with high drug loading and sustained drug release. To implement this idea, the anti-solvent co-precipitation of the HCPT and PEG-*b*-PLGA (monomethoxy polyethylene glycol-block-poly (lactide-co-glycolide)) will be employed under sonication to control the nucleation of NDs with nanocrystalline HCPT. In the fabrication, the core of HCPT will be wrapped with PEG-*b*-PLGA as steric stabilizers. In vitro studies will then be systematically carried out to examine the effect of the HCPT-loaded NDs against Hela cells.

## Methods

### Materials

All chemicals were analytical grade and used as received without further purification. The ultrapure water (18 MΩ∙cm-1) was used throughout the work. The 10-HCPT (purity >99 %) was purchased from Lishizhen Pharmaceutical Co., Ltd. The monomethoxy (polyethylene glycol)-poly (lactide-co-glycolide) (MPEG-PLA, PEG: 10 %, 5000 Da, PLA: 28000 Da, 85/15) was obtained from Daigang Biotechnology Co., Ltd.

### Preparation of the HCPT-Loaded NDs

The HCPT-loaded NDs were prepared via an ultrasound-assisted emulsion crystallization technique followed by the lyophilization treatment. Briefly, 5 mg HCPT and 5 mg PEG-*b*-PLGA were first co-dissolved in 5 mL acetone as the oil phase, which was then added dropwise into deionized water of 40 mL in volume in 5 min with sonication at the power of 200 W in an ice bath. Afterwards, the remaining organic solvent was removed by the rotary vacuum evaporation at 37 °C, and the resultant aqueous solution was filtered through a 1-μm cellulose acetate filter membrane to remove small drug nanocrystals as well as copolymer aggregates. The produced suspension was lyophilized for 24 h to get the dried powder.

### Preparation of the HCPT-Loaded NSs

The HCPT-loaded nanospheres (NSs) were prepared by a facile dialysis method. In brief, 100 mg of PEG-*b*-PLGA was dissolved in 10 mL of acetone (solution A), and 10 mg of HCPT was dissolved in 0.5 mL of 0.01 M NaOH solution (solution B). Then, solution B was dropped into solution A and the mixture was used as the organic phase. Subsequently, the resulting organic phase was then introduced into a dialysis bag and dialyzed against 1000 mL of water as the aqueous phase for 8 h. The as-prepared HCPT-loaded PEG-*b*-PLA nanoparticles (NPs) were lyophilized for 24 h using a freeze drier and stored at 4 °C for use.

### Preparation of the HCPT-Loaded NRs

The HCPT-loaded nanorods (NRs) were prepared via an emulsion crystallization technique followed by the lyophilization treatment. Briefly, 5 mg HCPT and 3 mg PEG-*b*-PLGA were first co-dissolved in 5 mL acetone as the oil phase, which was then added dropwise into deionized water of 40 mL in volume under vigorous stirring. Afterwards, the remaining organic solvent was removed by the rotary vacuum evaporation at 37 °C. The produced suspension was lyophilized for 24 h to get the dried powder.

### Characterization

The morphology of the HCPT-loaded NDs, NRs, and NSs was examined by SEM (UV-70) at 20 kV. The average particle size and size distribution of the NDs were determined by photon correlation spectroscopy with a Malvern Zetasizer Nano-ZS (Malvern Instruments, Malvern) at 25 °C under suitable dilution conditions. Measurements were repeated for three times to get the consistent results.

The amount of HCPT entrapped in the particles was determined indirectly by UV spectrophotometry (Beckman DU800). All samples were assayed at 383 nm. The weight of the drug entrapped in the particles was calculated by the calibration curve. Drug loading content and entrapment efficiency are presented by Eqs. (1) and (2):

1 $$ \mathrm{Drug}\kern0.5em \mathrm{loading}\kern0.5em \mathrm{content}\kern0.5em \left(\%\right)=\frac{\mathrm{weight}\kern0.5em \mathrm{of}\kern0.5em \mathrm{drug}\kern0.5em \mathrm{in}\kern0.5em \mathrm{N}\mathrm{P}\mathrm{s}}{\mathrm{weight}\kern0.5em \mathrm{of}\kern0.5em \mathrm{N}\mathrm{P}\mathrm{s}}\times 100\kern0.5em \% $$

2 $$ \mathrm{Entrapment}\kern0.5em \mathrm{efficiency}\kern0.5em \left(\%\right)=\frac{\mathrm{weight}\kern0.5em \mathrm{of}\kern0.5em \mathrm{drug}\kern0.5em \mathrm{in}\kern0.5em \mathrm{N}\mathrm{P}\mathrm{s}}{\mathrm{weight}\kern0.5em \mathrm{of}\kern0.5em \mathrm{feeding}\kern0.5em \mathrm{drug}}\times 100\kern0.5em \%. $$

The crystalline form of HCPT loaded in the NDs was analyzed using X-ray diffraction (XRD) (X'pert PRO). The X-ray diffractogram was scanned with Cu-ka radiation generated at 30 mA and 40 kV. The diffraction angle was increasing from 5 to 60°, with a step size of 0.016°.

### In Vitro Drug Release Studies

The in vitro drug release studies of particles were performed using the dialysis technique. The HCPT-loaded NDs were dispersed in phosphate-buffered saline (PBS, 10 mL) and placed into a pre-swelled dialysis bag (MWCO 3500 Da). The dialysis bag was then immersed in 0.1 M PBS at pH 7.4 and oscillated continuously in a shaker incubator (100 rpm) at 37 °C. All samples were assayed by fluorescence spectrophotometry. Free HCPT at the equivalent concentrations were used for comparison.

### Cellular Uptake Assay

Confocal imaging of cells was performed using a Leica laser scanning confocal microscope. Imaging of HCPT was carried out under 382-nm laser excitation, and emission was collected in the range of 528 nm. HeLa cells, MG-63 cells, MCF-7 cells, and MC3T3-E1 cells were incubated with HCPT-loaded NDs, NRs, and NSs ([HCPT] = 1 mg/mL, the concentration of both particles was determined by UV-vis measurement at 382 nm) for 0.5, 1.5, and 4.5 h before confocal imaging. All cells were washed twice with PBS before confocal imaging.

### Cytotoxicity of the NDs, NRs, and NSs

The cytotoxicity of the NDs, NRs, and NSs was determined by the MTT assay. Briefly, an adequate number of exponential phase HeLa cells, MG-63 cells, and MCF-7 cells was plated in quintuplicate in a 96-well flat-bottomed microplate and incubated for 24 h in the culture solution containing NDs, NRs, NSs, or free HCPT. In this study, 20 mL of MTT (Sigma) solution (5 mg/mL in PBS) was added in each well, and the plates were incubated at 37 °C for another 4 h. This was followed by the addition of 150 mL dimethylsulfoxide (DMSO), and the plate was agitated on a water bath chader at 37 °C for another 30 min. The absorbance at 570 nm was measured using a microplate reader (model 680; Bio-Rad).

## Results and Discussion

### Formulation and Characterization of the NDs

**Fig. 1:**
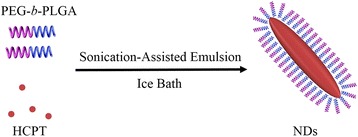
Schematic illustration of the preparation of NDs

**Fig. 2:**
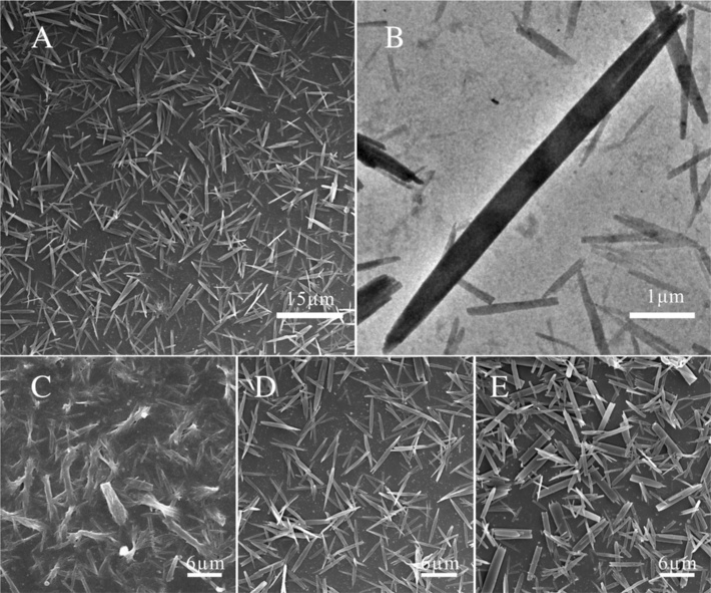
The SEM images and TEM images. **a** SEM image of the NDs. **b** TEM image of the NDs. And, SEM images of the NDs, when the ratio of HCPT to PEG-*b*-PLGA was 1:5 (**c**), 1:1 (**d**), and 2:1 (**e**)

The NDs were prepared by an ultrasound-assisted emulsion crystallization method as shown in Fig. 1. HCPT and PEG-*b*-PLGA were co-dissolved in acetone, forming a hybrid solution of drug and excipient. By injecting the hybrid solution into deionized water under sonication, the nucleation of HCPT nanocrystallines and the accompanying co-precipitation of PEG-*b*-PLGA onto the growing HCPT nanocrystallines presumably occurred due to a sudden change of solvent environment. Thus, the NDs with nanocrystalline HCPT as the core wrapped with PEG-*b*-PLGA as steric stabilizers were prepared successfully. We found that the ratio of HCPT to PEG-*b*-PLGA determined the morphology of the NDs, as shown in Fig. 2. When the ratio of the drug to the excipient was 1:5, excess PEG-*b*-PLGA may not only hinder the crystallization of HCPT, but also could not be fabricated into the NDs and deposited a layer (Fig. 2c). When increasing the amount of HCPT, NDs could be successfully manufactured (Fig. 2d). However, as the ratio was increased to 2:1, the rod-shaped particles could be seen in Fig. 1e, which were perhaps crystallized from the excess HCPT without the accompanying co-precipitation of the PEG-*b*-PLGA.

Figure 2a, d shows the needle-shaped morphology of the NDs with an average length of about 5 μm and a width of about 400 nm. This was in according with the TEM images (Fig. 2b). The result of DLS measurement shows a size of 356.7 nm (Additional file 1: Figure S1) and a zeta potential of −10.9 mv (Additional file 1: Figure S2). In order to investigate the drug loading of the NDs, ultraviolet spectroscopy was employed. It follows that the drug loading content was 15.04 % and the encapsulation efficiency was 30.08 %.

**Fig. 3:**
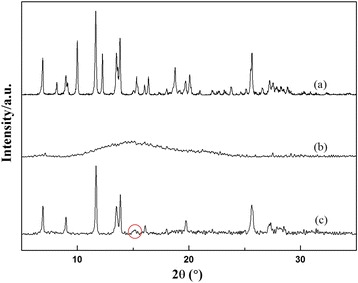
The XRD patterns of **a** free HCPT, **b** PEG-*b*-PLGA, and **c** HCPT-loaded NDs

Since the form of a drug is an important factor that affects the properties of the nanoparticles, it is highly important to understand the form of HCPT in the NDs. X-ray diffraction was employed to detect the form of HCPT within the NDs (Fig. 3). It is clear that pure HCPT shows many sharp crystalline peaks, representative of the characteristics of high crystallinity. On the other hand, a broad peak presents in PEG-*b*-PLGA, indicating that PEG-*b*-PLGA is in the amorphous state. As to the XRD pattern of the NDs, in addition to one broad and weak peak attributed to the semicrystalline PEG-*b*-PLGA, a number of sharp peaks of crystalline HCPT occur, the same as their bulk counterparts, suggesting its high crystallinity within the NDs. In short, the XRD results suggest that the growth kinetics of HCPT within the NDs had been changed, mainly due to the active occlusion and confining effects of PEG-*b*-PLGA.

### In Vitro Drug Release Studies

**Fig. 4:**
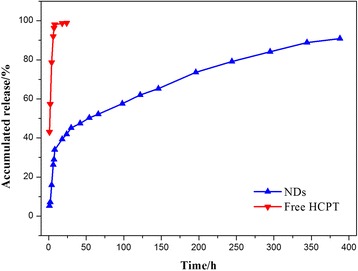
The drug release profile of free HCPT and HCPT-loaded NDs

The core-shell architecture of NDs plays an important role in the effect of sustained release of HCPT. The in vitro release studies of the NDs were performed using a dialysis technique, alongside free HCPT powders. All samples were assayed by fluorescence spectrophotometry (excitation at 382 nm). The release profiles are shown in Fig. 4. The profile of free HCPT showed that 40 % of the drug was released at the first sampling time of 1 h and almost 100 % by 8 h. The release profile of the NDs appears to have two components with a slight burst release of about 35 % at the sampling time of 8 h and followed by a remarkably prolonged release over the next 380 h. Although, the burst release still existed, it was reduced to a very low degree and the duration of sustained drug release had been prolonged to about 400 h. This can be attributed to the fact that a polymeric shell could limit the release of the drug in the core. These advantages could promote the application of the NDs for a sustained local drug delivery system.

### Cellular Uptake of the NDs by Cancer Cells

Confocal microscopy and fluorescence measurements were performed to assess the cellular uptake of NDs. Three types of cancer cells (HeLa cells, MG-63 cells, and MCF-7 cells) and one type of normal cell (MC3T3-E1 cells) were incubated with NDs for 0.5, 1.5, and 4.5 h, at 37 °C and then washed using PBS several times to remove the excess NDs: HCPT-loaded NSs with a drug loading of 7.0 % and a size distribution of 120.1 nm [49], collected from the dialysis method (see in the SI materials), and HCPT-loaded NRs with a drug loading of 35.2 % and a size distribution of 140 nm, collected from the emulsion crystallization method were used for comparison. The NSs and the NRs were prepared with the same materials—HCPT and PEG-*b*-PLGA—and thus they were detected to be with the similar properties such as the zeta potential. This was also found to affect the cellular uptake to a large degree. With a size of 120 nm (Additional file 1: Figure S3), the NSs were much smaller than the NDs and the NRs (Additional file 1: Figure S4), which should have been taken up much faster and much more than the two larger particles [12–17]. But, the result did not come out as expected.

**Fig. 5:**
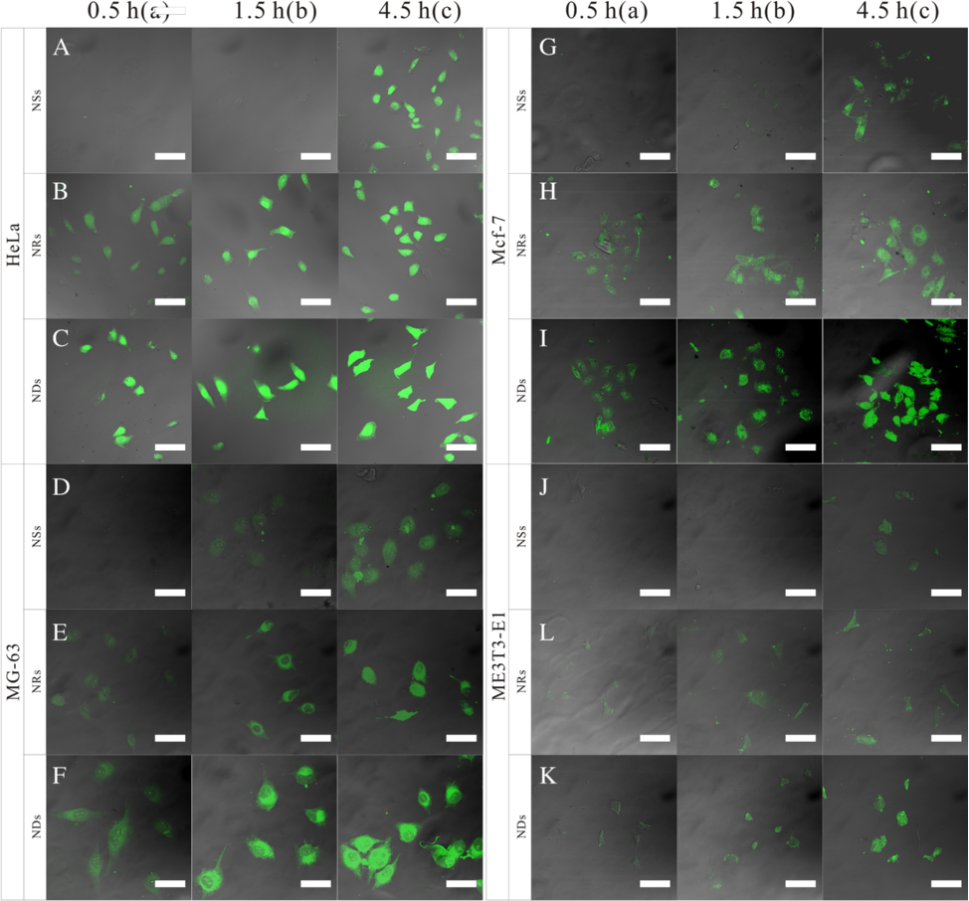
The CLSM images. The HeLa cells, MG-63 cells, MCF-7 cells, and MC3T3-E1 cells incubated with HCPT-loaded NSs ([HCPT] = 1 mg/mL), HCPT-loaded NRs ([HCPT] = 1 mg/mL), and NDs ([HCPT] = 1 mg/mL) for 0.5 h *(a)*, 1.5 h *(b)*, and 4.5 h *(c)* at 37 °C, respectively. All images were taken under identical instrumental conditions and presented at the same intensity scale. All *scale bars* are 25 μm

It follows from Fig. 5 that a much more intense fluorescence emission of HCPT was detected from all the four types of cells exposed to the NDs and the NRs than that of those exposed to NSs for the same sampling time. As shown in Fig. 5b, c, a relatively intense fluorescence of HCPT was detected from the HeLa cells exposed to the NDs and the NRs for 0.5 h. And, the intensity of fluorescence increased with the incubate time, indicating that the uptake of the NDs and the NRs began before the first sample time of 0.5 h, and amounts of NDs had been internalized into the HeLa cells at 0.5 h. But, as to the HeLa cells exposed to the NSs, no fluorescence could be detected under the same intensity of the excitation light at 0.5 and 1.5 h (Fig. 5a). Hence, the intensity of the excitation light was raised, and a weak fluorescence appeared (Additional file 1: Figure S5 A and B). The result indicates that very few NSs were internalized by the HeLa cells after incubating with the particles for 0.5 and 1.5 h. But, a relatively strong fluorescence emission was detected at the sample time of 4.5 h (Fig. 5a), illustrating that more NSs were internalized into the HeLa cells. Hence, the uptake of the majority of NSs by the HeLa occurred between 1.5 and 4.5 h, which was much later than that of the NDs and the NRs. Moreover, in comparing Fig. 5a with Fig. 5b, c, the fluorescence detected from the group of 0.5 h NDs and NRs incubation was stronger than that of 4.5 h NSs incubation, indicating that the amount of NDs and the NRs internalized into the HeLa cells was much more than that of the NSs. As to the MG-63 cells, MCF-7 cells, and MC3T3-E1 cells, identical results were acquired; the fluorescence emissions detected from the cells incubated with the NDs and the NRs were much earlier and stronger than that of the cells incubated with the NSs (Fig. 5c–h). These results distinctly demonstrated that a high-aspect ratio can increase the abundance and the rate of cellular uptake of the particles. And, this factor might play a more important role than the size of the particle.

Comparing Fig. 5b, and Fig. 5c, the fluorescence emission of HCPT from the cells exposed to the NDs was a little stronger than that of those exposed to the NRs at each simple time. With a similar aspect ratio, it might be the pointed end that influences the internalization of the particles. Furthermore, when normal cells (MC3T3-E1 cells) were incubated with the particles, the intensity of the fluorescence signal was much weaker than that of the cancer cells. This was probably because the membrane fluidity of the cancer cells were elevated, which would facilitate the internalization of the particles.

The drug amount difference in cellular uptake among the NRs, NDs, and NSs could be attributed to both the released drug and drug trapped inside the nanoparticles, no matter how much the ratio of PEG-*b*-PLGA to HCPT is. The cellular uptake time was designed between 0.5 and 4.5 h, in which a little amount of the drug was released (seen from the drug release profile), so the drug taken up in the cell was much due to the drug inside the nanoparticles. In addition, the drug release profile of NRs was not significantly different from that of the NDs (See Additional file 1: Figure S6). So, the difference is mainly attributed to the shapes of nanoparticles instead of the released drug from the particles or the ratio of PEG-*b*-PLGA to HCPT.

The process in which the drug was first released into the extracellular medium and diffused into cells truly happened in the experiment. To further verify the role of the released drug property in cellular uptake, free HCPT was used as control in the cellular uptake experiment. But just as mentioned above, the longest incubation time was only 4.5 h, during which the amount of released drug was <20 %. Hence, we used free HCPT (the concentration was 20 % of that of the NDs) as control. Since a much more intense fluorescence signal of HCPT was detected from the HeLa cells exposed to the NDs than that of those exposed to free HCPT (See Additional file 1: Figure S7), the fluorescence in the HeLa cells was mainly caused by the internalization of the NDs, rather than that of HCPT. In effect, the different shapes of nanoparticles in the extracellular medium were just like cocci or bacillus with a little bit of soft property. Once the nanoparticles were taken up by the cells, the particles were immediately swallowed by the lysosome,in which the different shapes of particles were all decomposed into fragments by a variety of decomposition enzymes.

**Fig. 6:**
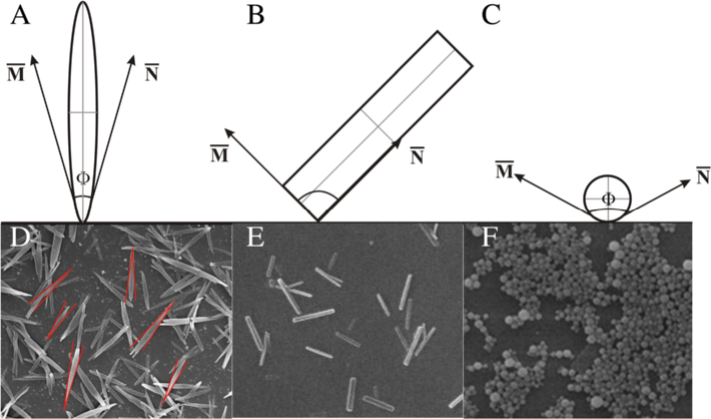
Definition of *Φ* and the schematic illustration of the internalization of NDs, NRs, and NSs (**a**–**c**). $\overline{M}$ and $\overline{N}$ are vectors that show the flow directions of the cell membranes. *Φ* is the angle between $\overline{M}$ and $\overline{N}$. **d** The measurement of the *Φ* of the NDs. **e**–**f** The SEM of the NRs and NSs

Taking the size effect into account, the NSs with a much smaller size should have been much more favored in the cellular internalization process. However, the opposite was observed; the confocal laser scanning microscopy (CLSM) images further validate that the cellular internalization of the nanoparticles exhibits a strong shape dependence, which may be stronger than the influence of size. The results demonstrate the following fact: the high-aspect-ratio nanoparticles are internalized much more rapidly and efficiently than those with the equal aspect ratio [48]. Moreover, the phagocytosis may also be in favor of the particles with sharp ends. Champion JA et al. found that a macrophage attached to an ellipse at the pointed end will internalize the particle in a few minutes, while a macrophage attached to a flat region of the same ellipse will not internalize it for over 12 h. Here, we define the angle Φ to roughly illustrate the role of shape in non-phagocytosis uptake (Fig. 6). $\overline{\mathrm{M}}$ and $\overline{\mathrm{N}}$ are vectors that show the flow directions of the cell membranes. Φ is the angle between $\overline{\mathrm{M}}$ and $\overline{\mathrm{N}}$. The cell membranes flow easily, when the Φ is small. The Φ of the NDs is 10.19 ± 1.47° (Fig. 6b), much smaller than 90° of the NDs. When the NDs were attached to the HeLa cells at the sharp end, they would be taken up in a short time, mainly less than 0.5 h. This explains the fact that Fig. 5d shows a more intense fluorescent emission. When the NDs attached to the HeLa cells at the flat region, they would change the orientation until being taken up. As to the NRs, their internalization began from the vertexes and the Φ was 90°, which took more time to complete the uptake. And, the Φ of the NSs was much larger than 90°, which took the longest time.

Notably, it was believed that the upper limit of the size that can be taken up into non-phagocytic cells was 150 nm. In the study herein, however, the internalization of NDs with an average length between 5 μm was clearly observed in CLSM images. Therefore, the particle size, measured simply by diameter for spheres, must have a new definition to non-spherical particles since they may have two or more different length scales. And, the new definition should take into account the end, the angle, the asymmetry, and some other factors of the non-spherical particles to make it more accurate.

### Cytotoxicity of the NDs, NRs, and NSs

To further investigate the possibility of utilizing the NDs for local drug delivery, we tested the killing ability of the NDs to cancer cells. The cytotoxicity of NDs, NRs, NSs, and free HCPT was evaluated using the MTT assay with the HeLa cells, MG-63 cells, and MCF-7 cells. The NDs, NSs, and free HCPT containing the equivalent concentrations of HCPT were, respectively, incubated with the three types of cells for 24 and 48 h.

**Fig. 7:**
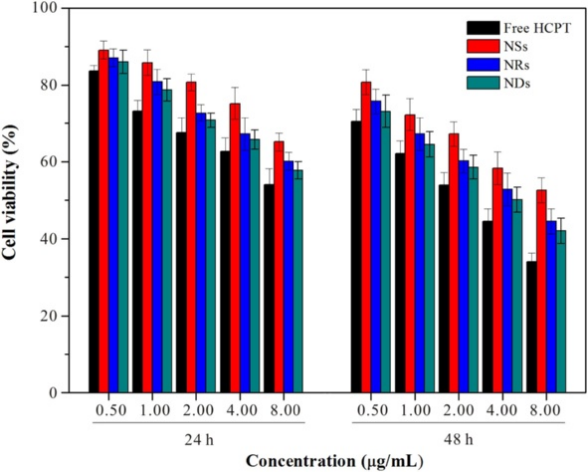
The in vitro cytotoxicity assay against the HeLa cells, *p* < 0.05

It shows in Fig. 7 that the NDs and the NRs reveal a much higher degree of toxicity to the HeLa cells than that of the NSs at 24 h at equivalent concentrations of HCPT. The possible explanation is as follows. Firstly, because of the enhanced cellular uptake, more NDs and NRs could be taken up by the HeLa cells, and release more HCPT in the cells, which kills the cells instantly. Secondly, for its high drug loading, the NDs and the NRs have a slight burst release at 8 h, resulting in the fact that the HCPT concentration in the group of NDs was higher than that of the NSs both in and out of the cells. And, the difference of the cell viability between the group of NDs and NSs became more obvious at 48 h, owing to the fact that the influence of the above two factors became larger when the incubated time was prolonged to 48 h. Meanwhile, we could still find that the NDs’ toxicity was higher than that of the NRs’, which might be owing to the enhanced cellular uptake of the NDs caused by the sharp end. Compared with the three kinds of NPs, free HCPT shows the highest inhibition rate, mainly due to the fact that free HCPT could increase the drug concentration to a high level within a short time and directly acted on the cells. As to the other two types of cells, although their sensitivity to HCPT was different from the HeLa cells, the order of the killing ability was free HCPT > NDs > NRs > NSs (Additional file 1: Figure S6). This was in accordance with the result of the HeLa cells.

## Conclusions

The study herein presents a simple approach to obtain the HCPT-loaded, high-aspect- ratio, pointed-end NDs with high drug loading content and sustained drug release. The CLSM demonstrates the more effective cellular internalization of NDs than that of nanospheres with a much smaller size. The MTT experiment indicated that the NDs show an enhanced cytotoxicity to the three types of cells. Although the NDs are too large to be used as systemic injections, the NDs are promising as a sustained local drug delivery system for the treatment of tumor for their easier internalization and high cytotoxicity to the cancer cells. More importantly, the fabrication of NDs opens a door to design new formulations of nanoneedle drug delivery systems, or at least nanorod drug delivery systems, which are more efficient in cancer chemotherapy compared with the spherical nanodrug delivery system.

## Additional File

Additional file 1: Figure S1. The size distribution of HCPT-loaded NDs. **Figure S2.** The Zeta Potential of HCPT-loaded NDs. **Figure S3.** The SEM images of HCPT-loaded NSs. **Figure S4.** The SEM images of HCPT-loaded NRs. **Figure S5.** The CLSM images at higher intensity scale. HeLa cells incubated with HCPT-loaded NSs ([HCPT] = 1 mg/mL) for A) 0.5 h and B) 1.5 h. C) MG-63 cells incubated with HCPT-loaded NSs ([HCPT] = 1 mg/mL) for 0.5 h. D) MCF-7 cells incubated with HCPT-loaded NSs ([HCPT] = 1 mg/mL) for 0.5 h. MC3T3-E1 cells incubated with HCPT-loaded NSs ([HCPT] = 1 mg/mL) for E) 0.5 h and F) 1.5 h. **Figure S6.** In vitro drug release profiles of NDs, NRs and NSs in PBS (pH 7.4) at 37 °C. **Figure S7.** The CLSM images. The HeLa cells incubated with (A) free HCPT ([HCPT] = 0.2 mg/mL), and (B) NDs ([HCPT] = 1 mg/mL) for 4.5 h at 37 °C. All images were taken under identical instrumental conditions and presented at the same intensity scale. All scale bars are 25 μm. **Figure S8.** The in vitro cytotoxicity assay against the MG-63 cells (A) and the MCF-7 cells (B), *p* < 0.05. (DOC 1389 kb)
