# Supplementary material for: Preparation of HCPT-Loaded Nanoneedles with Pointed Ends for Highly Efficient Cancer Chemotherapy
Source: Nanoscale Res Lett. 2016 Jun 14;11:294. doi: 10.1186/s11671-016-1491-9 (PMC4907965; doi:10.1186/s11671-016-1491-9)
Supplement: Additional file 1: Figure S1. — The size distribution of HCPT-loaded NDs. Figure S2. The Zeta Potential of HCPT-loaded NDs. Figure S3. The SEM images of HCPT-loaded NSs. Figure S4. The SEM images of HCPT-loaded NRs. Figure S5. The CLSM images at higher intensity scale. HeLa cells incubated with HCPT-loaded NSs ([HCPT] = 1 mg/mL) for A) 0.5 h and B) 1.5 h. C) MG-63 cells incubated with HCPT-loaded NSs ([HCPT] = 1 mg/mL) for 0.5 h. D) MCF-7 cells incubated with HCPT-loaded NSs ([HCPT] = 1 mg/mL) for 0.5 h. MC3T3-E1 cells incubated with HCPT-loaded NSs ([HCPT] = 1 mg/mL) for E) 0.5 h and F) 1.5 h. Figure S6. In vitro drug release profiles of NDs, NRs and NSs in PBS (pH 7.4) at 37 °C. Figure S7. The CLSM images. The HeLa cells incubated with (A) free HCPT ([HCPT] = 0.2 mg/mL), and (B) NDs ([HCPT] = 1 mg/mL) for 4.5 h at 37 °C. All images were taken under identical instrumental conditions and presented at the same intensity scale. All scale bars are 25 μm. Figure S8. The in vitro cytotoxicity assay against the MG-63 cells (A) and the MCF-7 cells (B), p < 0.05. (DOC 1389 kb) [file 11671_2016_1491_MOESM1_ESM.doc]

**Figure S1.** The size distribution of HCPT-loaded NDs.

**Figure S2.** The Zeta Potential of HCPT-loaded NDs.


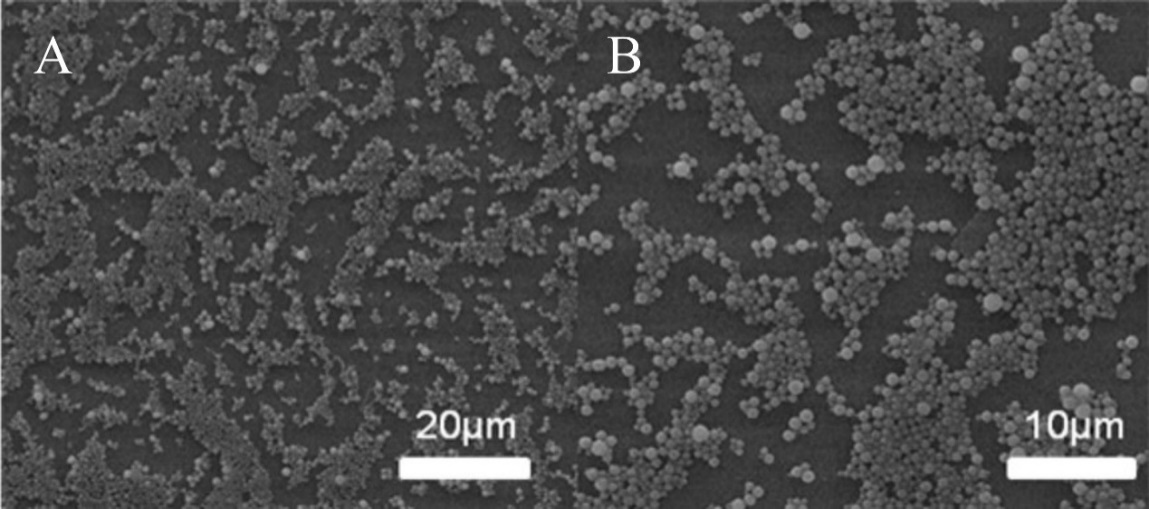


**Figure S3.** The SEM images of HCPT-loaded NSs.


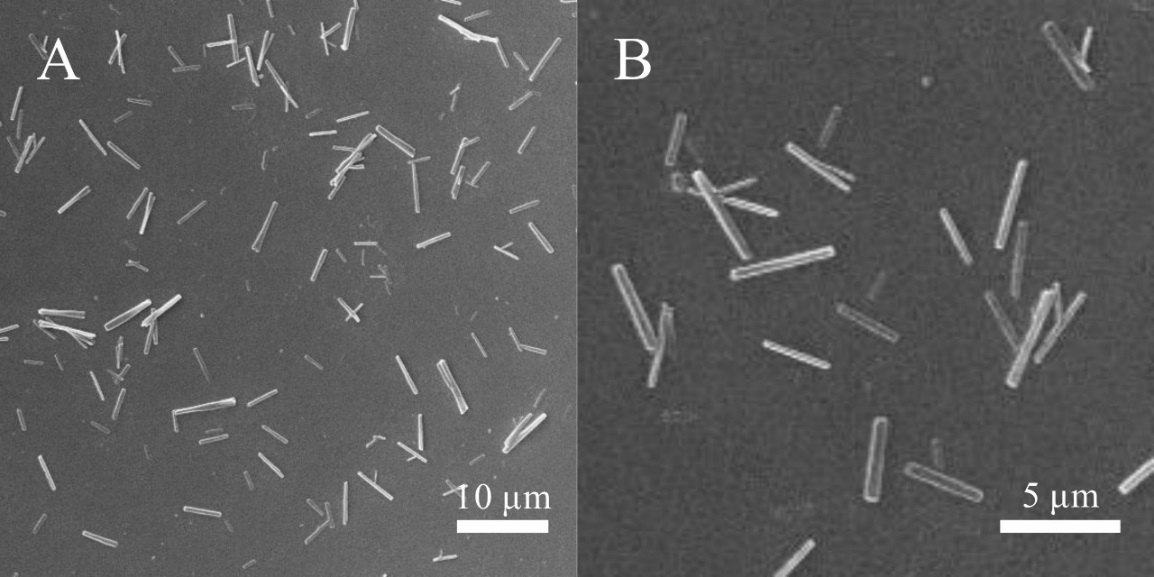


**Figure S4.** The SEM images of HCPT-loaded NRs


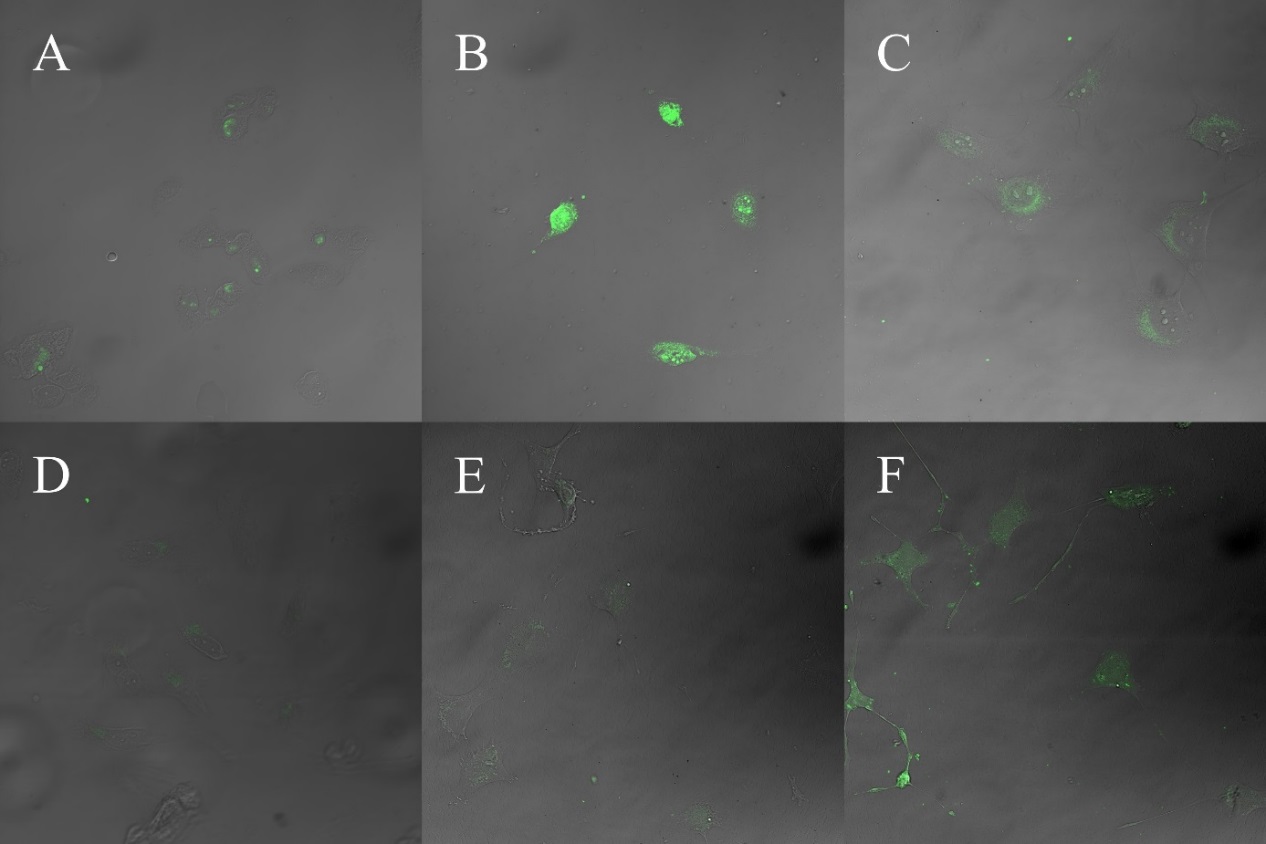


**Figure S5.** The CLSM images at higher intensity scale. HeLa cells incubated with HCPT-loaded NSs ([HCPT] = 1 mg/mL) for A) 0.5 h and B)1.5 h. C) MG-63 cells incubated with HCPT-loaded NSs ([HCPT] = 1 mg/mL) for 0.5 h. D) MCF-7 cells incubated with HCPT-loaded NSs ([HCPT] = 1 mg/mL) for 0.5 h. MC3T3-E1 cells incubated with HCPT-loaded NSs ([HCPT] = 1 mg/mL) for E) 0.5 h and F)1.5 h.


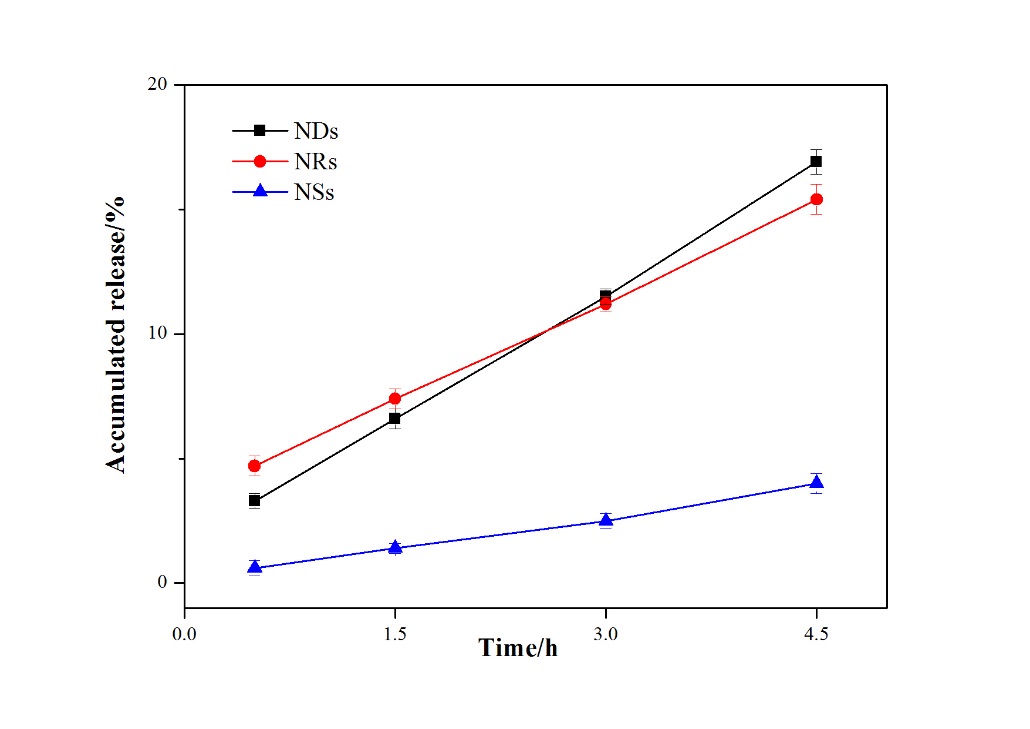


**Figure S6.** In vitro drug release profiles of NDs, NRs and NSs in PBS (pH 7.4) at 37 ºC.


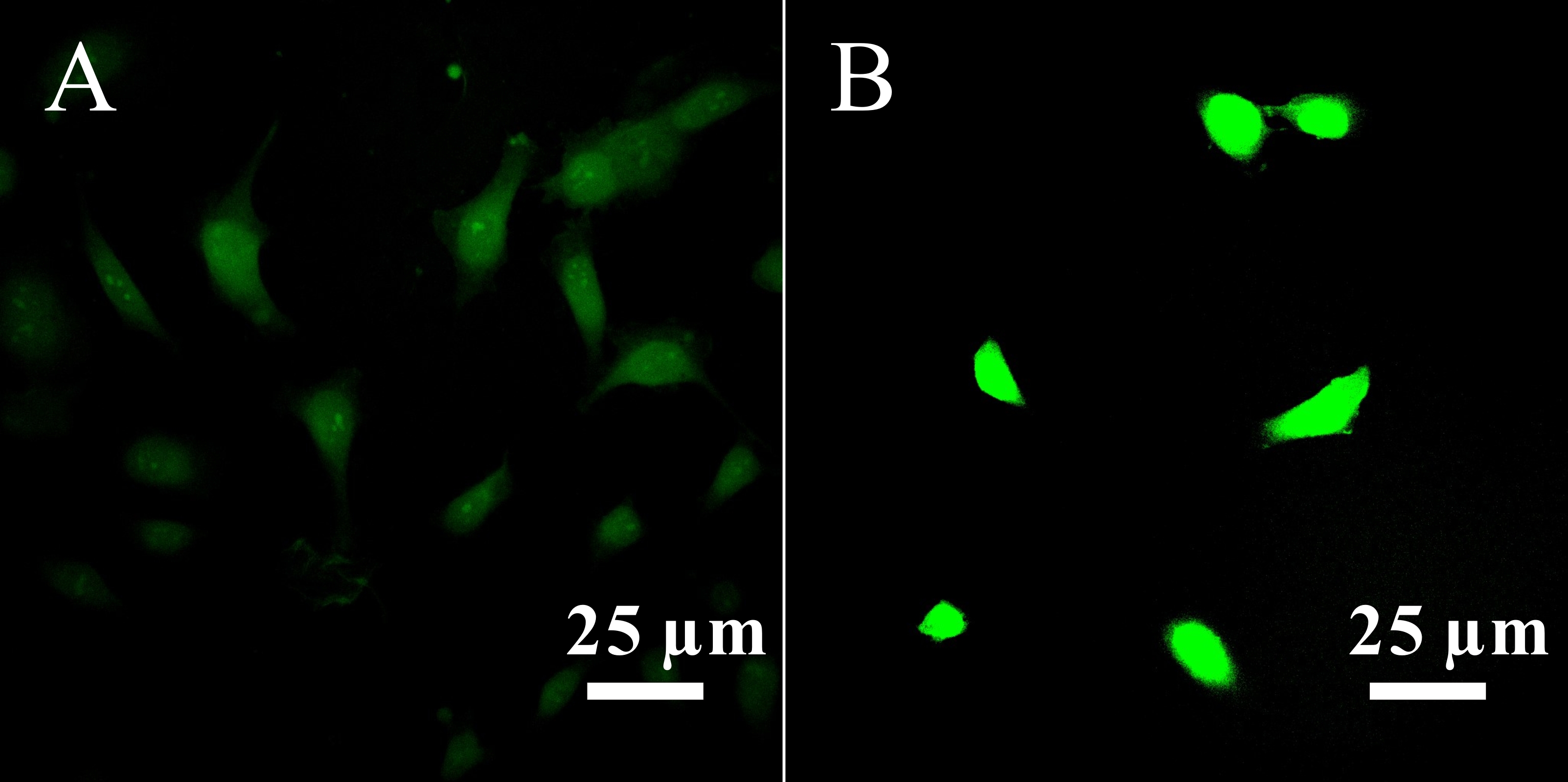


**Figure S7.** The CLSM images. The HeLa cells incubated with (A) free HCPT ([HCPT] = 0.2 mg/mL), and (B) NDs ([HCPT] = 1 mg/mL) for 4.5 h at 37 ºC. All images were taken under identical instrumental conditions and presented at the same intensity scale. All scale bars are 25 μm.


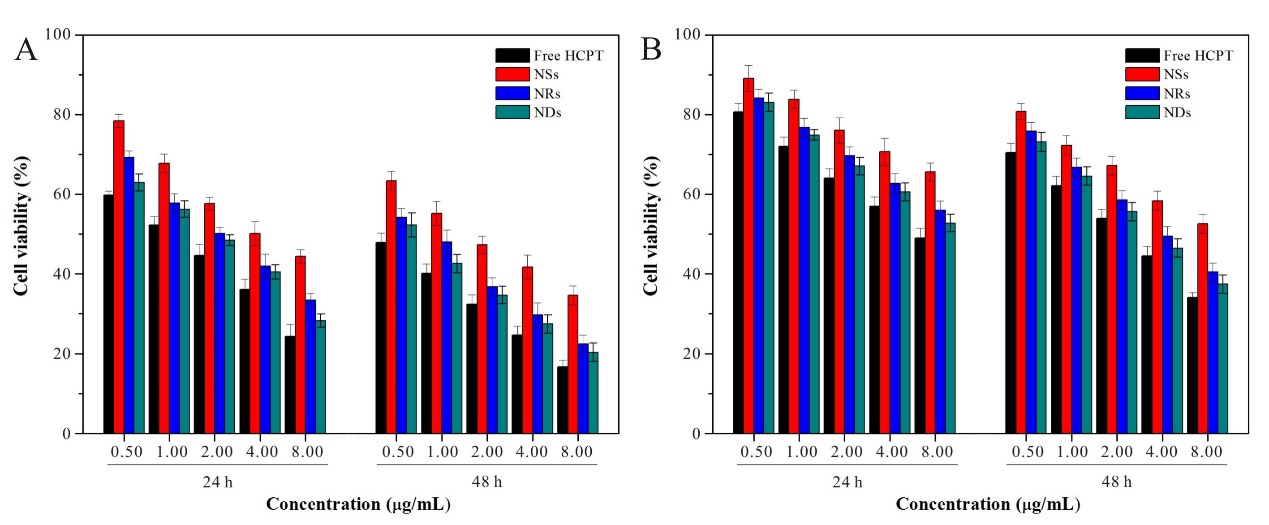


**Figure S8.** The in vitro cytotoxicity assay against the MG-63 cells (A) and the MCF-7 cells (B), p < 0.05.
